# Preparation and Thermal Conductivity of Epoxy Resin/Graphene-Fe_3_O_4_ Composites

**DOI:** 10.3390/ma14082013

**Published:** 2021-04-16

**Authors:** Zhong Wu, Jingyun Chen, Qifeng Li, Da-Hai Xia, Yida Deng, Yiwen Zhang, Zhenbo Qin

**Affiliations:** 1 Tianjin Key Laboratory of Composite and Functional Materials, School of Material Science and Engineering, Tianjin University, Tianjin 300072, China; wuzhong2319@163.com (Z.W.); Jingyun19961229@163.com (J.C.); qifengli@tju.edu.cn (Q.L.); dahaixia@tju.edu.cn (D.-H.X.); yida.deng@tju.edu.cn (Y.D.); ywzsci@tju.edu.cn (Y.Z.); 2 Key Laboratory of Advanced Ceramics and Machining Technology (Ministry of Education), Tianjin University, Tianjin 300072, China

**Keywords:** magnetic GR, EP/GR-Fe_3_O_4_ composites, orientation, thermal conductivity

## Abstract

By modifying the bonding of graphene (GR) and Fe_3_O_4_, a stable structure of GR-Fe_3_O_4_, namely magnetic GR, was obtained. Under the induction of a magnetic field, it can be orientated in an epoxy resin (EP) matrix, thus preparing EP/GR-Fe_3_O_4_ composites. The effects of the content of GR and the degree of orientation on the thermal conductivity of the composites were investigated, and the most suitable Fe_3_O_4_ load on GR was obtained. When the mass ratio of GR and Fe_3_O_4_ was 2:1, the thermal conductivity could be increased by 54.8% compared with that of pure EP. Meanwhile, EP/GR-Fe_3_O_4_ composites had a better thermal stability, dynamic thermomechanical properties, and excellent electrical insulation properties, which can meet the requirements of electronic packaging materials.

## 1. Introduction

Epoxy resin (EP) sealing materials are widely used in the field of electronic packaging due to their excellent electrical insulation and mechanical properties. However, the heat-conducting property of EP is very poor, and the intrinsic thermal conductivity is only 0.18 W/mK [1]. With the development of miniaturization of electronic equipment, the phenomenon of heat generation becomes more and more serious, which will greatly shorten the service life of the equipment [2]. Therefore, improving the thermal conductivity of EP is an efficient technical means to solve this problem. At present, several conductive particles (SiC [3,4], Al_2_O_3_ [5,6,7], AlN [8,9,10,11], and SiO_2_ [12]) are used to improve the thermal conductivity of EP. However, the improvement effect is not obvious. To effectively improve the heat dissipation rate of EP, the suggestion of constructing a heat conduction channel by adding graphene (GR) to an EP matrix has been proposed. Because of the extremely high thermal conductivity of GR in the in-plane direction, it is crucial to ensure the directional distribution of GR in an EP matrix. There are many methods to achieve directional distribution of GR in an matrix, including the flow shear induction method [13], solution pouring method [14], vacuum dead-end microporous filtration method [15], vacuum pumping filtration method [16], and the magnetic field induction method [17]. The magnetic field induction method refers to the directional arrangement of magnetic GR in a magnetic field, which is widely used because of its safety and simple operation. Therefore, the preparation of magnetic GR is a goal that researchers have been exploring. Zhu successfully loaded magnetic nanoparticles on the surface of GR via chemical co-precipitation [18]. Ma prepared GR loaded with Fe_3_O_4_ nanoparticles using a decomposition reduction method [19]. Guo used polyelectrolyte coating technology to coat Fe_3_O_4_ nanoparticles on the surface of GR [20]. Although magnetic particles can be loaded on the surface of GR using different methods, this structure is not sufficiently stable.

In this paper, magnetic GR with a stable structure was obtained using a chemical bonding method. The directional distribution of GR in an EP matrix was achieved under the induction of a magnetic field. Based on the high thermal conductivity of GR in the in-plane direction, an excellent thermal conductivity of EP/GR-Fe_3_O_4_ composites was realized.

## 2. Materials and Methods

### 2.1. Materials

Graphene nanosheets were purchased from Nanjing Xianfeng Nanotechnology Co., Ltd., Nanjing, China. NH_2_C_3_H_6_Si(OC_2_H_5_)_3_ (KH550) and C_9_H_20_O_5_Si (KH560), used as the silane coupling agent with the amino group (NH_2_) and epoxy group, respectively, were purchased from Aladdin Chemistry Co., Tianjin, China. FeCl_3_·6H_2_O and FeCl_2_·4H_2_O were provided by Tianjin Yuanli Chemical Co., Ltd., Tianjin, China. Bisphenol-A liquid epoxy resin (E51), phenolic amine epoxy curing agent (T31), 2,4,6-Tris (dimethylamino methyl) phenol curing accelerator (DMP-30) and epoxy resin defoamer were purchased from Nantong Xingchen Synthetic Material Co., Tianjin, China. Concentrated hydrochloric acid (HCl), sodium hydroxide (NaOH) and ethanol were purchased from Tianjin Yuanli Chemical Co., Ltd., Tianjin, China.

### 2.2. Preparation

Firstly, GR (0.3 g) was dispersed in ethanol (60 mL), then KH550 (3 mL) and HCl (1 mL) were added. The mixed solution was stirred in an oil bath at 55 °C for 5 h to obtain GR-NH_2_ after suction filtration and ultrasonic dispersion. FeCl_3_·6H_2_O (2.0 g) and FeCl_2_·4H_2_O (0.74 g) were dissolved in deionized water (20 mL). An appropriate amount of NaOH was added to make the pH value of the solution within the range of 10–11, and the mixed solution was stirred in an oil bath at 55 °C for 2 h [21]. Then, the intermediate products obtained by filtering the above solution were added to a mixed solution of ethanol (30 mL), KH560 (1 mL) and HCl (1 mL), and stirred in an oil bath at 55 °C for 5 h. After filtration, the Fe_3_O_4_-epoxy group was obtained. Finally, GR-NH_2_ and Fe_3_O_4_-epoxy (mass ratios are 8:1, 4:1, 2:1, respectively) were added into ethanol (60 mL) and stirred in an oil bath at 60 °C for 10 h. The mixed solution was filtered to obtain magnetic GR.

E51 (8 g), T31 (2 g), DMP-30 (0.16 g) and epoxy resin defoamer (0.08 g) were added to the polytetrafluoroethylene (PTFE) mold. A certain amount of magnetic GR (the specific content is shown in Table 1) was added to the EP mixture, and the magnetic GR was evenly dispersed in the EP mixture through stirring. The mold containing a mixture of EP and magnetic GR was placed in a uniform magnetic field and stood for 24 h until the mixture was cured [22]. As described above, magnetic GRs with mass ratios of GR to Fe_3_O_4_ of 8:1, 4:1, and 2:1 were prepared, numbered a, b, and c. When the mass fraction of GR in the EP was maintained as 0.034 wt.%, 0.067 wt.%, 0.100 wt.%, and 0.134 wt.%, the corresponding addition amount of magnetic GR (a, b, c) in the EP is shown in Table 1.

### 2.3. Characterization

The morphology of samples was characterized by scanning electron microscopy (SEM, JSM, 7800 F, Shishima, Tokyo, Japan) and transmission electron microscope (TEM, JEM, 2100 F, Shishima, Tokyo, Japan), and the elements were determined by the energy dispersive spectroscopy (EDS, AMETEXK EDAX, Shishima, Tokyo, Japan). The crystal structure of materials was determined by X-ray diffraction (XRD, D8 ADVANCE, Shishima, Tokyo, Japan) with Cu Kα radiation. The microstructure of the magnetic-particle was determined by Fourier transform infrared spectrometer (FTIR, Nicolet iS10, Waltham, MA, USA). The thermal conductivity was measured by a thermal constant analyzer (TPS2500S, Hot Disk, Uppsala, Sweden). Dynamic thermomechanical analysis (DTMA) of the EP and EP/GR-Fe_3_O_4_ composites were determined by TA Instrument Thermal Mechanical Analyzer (TMA, Q800, New Castle, DE, USA). Thermo-gravimetric analysis (TGA) was carried out with a synchronous thermal analyzer (STA449, Selbu, Germany). The volume resistivity of EP and its composites were measured with an ultrahigh electric resistivity meter (ZC36, Shanghai, China). The test voltages for the samples were 250 V and 1000 V.

## 3. Results and Discussion

### 3.1. Material Characterization

**Figure 1:**
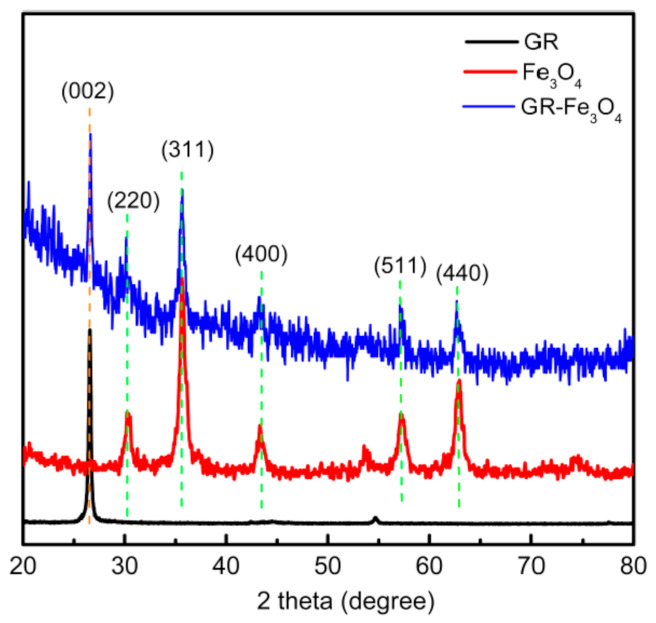
XRD patterns of GR, Fe_3_O_4_ and magnetic GR (GR-Fe_3_O_4_).

Fe_3_O_4_ is a black crystal with magnetism, commonly known as magnetic iron oxide, which is a complex oxide. The ferromagnetic material prepared has typical Fe_3_O_4_ diffraction peaks. As shown in Figure 1, the positions at 30.1°, 35.4°, 43.1°, 56.9°, and 62.5° correspond to the diffraction peaks (220), (311), (400), (511), (440) of Fe_3_O_4_, respectively [21]. GR has a characteristic peak of (002) at 26.5°. Additionally, the prepared magnetic GR was also characterized by XRD, and it was found that the characteristic peaks of GR and Fe_3_O_4_ existed at the same time, indicating that GR-Fe_3_O_4_ was successfully prepared. To determine whether Fe_3_O_4_ and GR was chemically bonded, FTIR characterization was performed.

**Figure 2:**
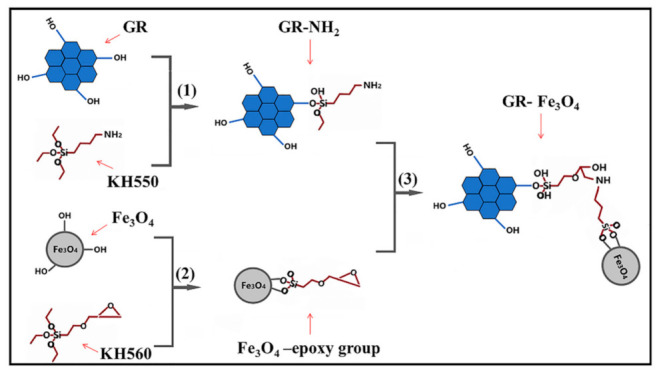
Schematic diagram of magnetic GR preparation.

**Figure 3:**
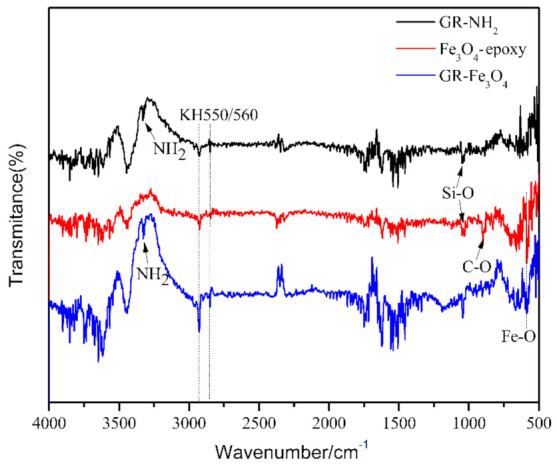
FTIR spectra of GR-NH_2_, Fe_3_O_4_-epoxy and GR-Fe_3_O_4_.

Figure 2 shows the corresponding preparation schematic diagram. Steps (1), (2), and (3) are the preparation principles of GR-NH_2_, Fe_3_O_4_-epoxy, and magnetic GR, respectively. Step (1): the KH550 with NH_2_ reacts with the hydroxyl groups of the GR, so that the GR is loaded with NH_2_. Step (2): the Fe_3_O_4_ with hydroxyl groups reacts with KH560 with epoxy groups to make Fe_3_O_4_ have epoxy groups. Step (3): through the reaction between the NH_2_ and epoxy groups, the magnetic particles Fe_3_O_4_ are attached to the GR. Figure 3 shows the FTIR spectra of GR-NH_2_, Fe_3_O_4_-epoxy and GR-Fe_3_O_4_. In the FTIR spectrum, the stretching vibration peak of Si-O is at 1043 cm^−1^ [23]. The absorption peak at 3326 cm^−1^ corresponds to the stretching vibration peak of NH_2_. The antisymmetric stretching vibration peaks of methylene on KH550 and KH560 are at 2920 cm^−1^ and 2850 cm^−1^ [24]. The characteristic peak of Fe-O is at 580 cm^−1^ [25,26,27,28], and the characteristic peak of epoxy group C-O is at 890 cm^−1^ [29]. As shown in Figure 3, stretching vibration peaks of Si-O and NH_2_ appears after the reaction of GR and KH550, indicating that GR-NH_2_ is successfully prepared. After Fe_3_O_4_ reacts with KH560, the characteristic peak of epoxy group C-O appears, as shown in Figure 3, indicating the successful preparation of Fe_3_O_4_-epoxy. When the Fe_3_O_4_-epoxy reacts with the GR-NH_2_, the absorption peak around 3326 cm^−1^ becomes wider, and the characteristic peak of the epoxy group C-O at 890 cm^−1^ disappears, indicating the successful preparation of magnetic GR.

**Figure 4:**
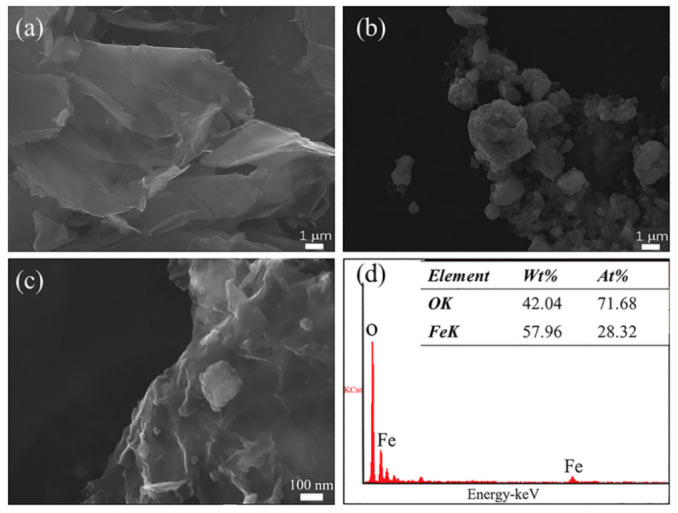
SEM images of (**a**) GR, (**b**) Fe_3_O_4_, (**c**) GR-Fe_3_O_4_; (**d**) the EDS of GR-Fe_3_O_4_.

Figure 4 shows the SEM morphology of GR, Fe_3_O_4_, GR- Fe_3_O_4_, and EDS of GR-Fe_3_O_4_. The surface of GR has an obvious fold structure, as shown in Figure 4a. Figure 4b shows the SEM morphology of Fe_3_O_4_. Since the SEM resolution is lower than TEM, it is impossible to determine whether it is a single Fe_3_O_4_ particle. There are many small particles attached to the GR, as shown in Figure 4c. EDS characterization test was performed on the particles on magnetic GR, which is depicted in Figure 4d. Element peaks associated with iron and oxygen are detected, indicating that Fe_3_O_4_ particles loaded on the GR sheet.

**Figure 5:**
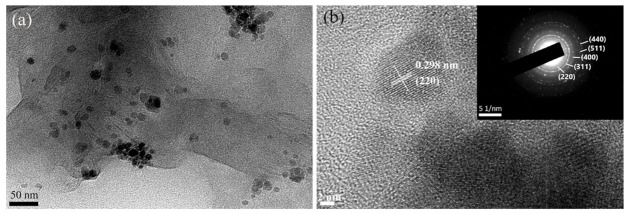
TEM image (**a**) and electron diffraction pattern (**b**) of GR-Fe_3_O_4_.

The prepared magnetic GR was further observed by TEM. As shown in Figure 5, Fe_3_O_4_ particles are evenly distributed on the GR flakes. The ones with lattice fringes at high magnification are Fe_3_O_4_, and the diameter of a single crystal grain is in the range of 15–20 nm. The electron diffraction patterns are (220), (311), (400), (511), and (440), respectively, indicating that the chemical bonding of GR and Fe_3_O_4_ did not change the crystal structure of the Fe_3_O_4_ particles.

**Figure 6:**
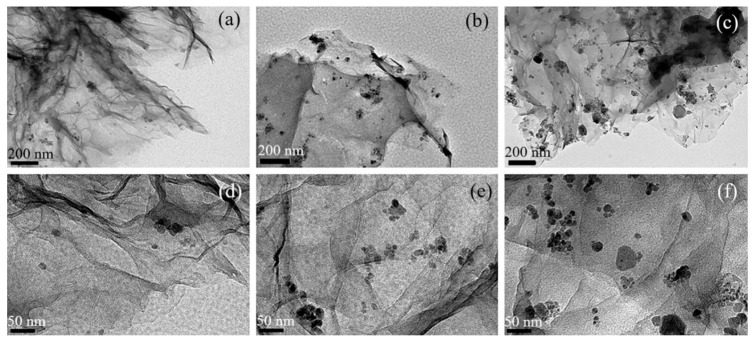
TEM images of GR-Fe_3_O_4_ under different mass ratios, the mass ratios of GR: Fe_3_O_4_ are 8:1 (**a**,**d**), 4: 1 (**b**,**e**), 2:1 (**c**,**f**).

The mass ratios of GR and Fe_3_O_4_ are 8:1, 4:1, 2:1, respectively. According to the different loading content of Fe_3_O_4_ on GR, the orientation degree of GR in the magnetic field is also different. It is expected that the more loading of Fe_3_O_4_ content, the more horizontal and oriented the distribution of GR in EP will be, which will significantly improve the thermal conductivity. TEM characterization of GR with different loadings of Fe_3_O_4_ is shown in Figure 6. The macro morphology observed at 15k magnification (Figure 6a–c) indicates more Fe_3_O_4_ loaded on GR with an increase in the mass ratio of Fe_3_O_4_: GR. However, from the observation at 50 k magnification (Figure 6d–f), it is found that Fe_3_O_4_ has the best dispersibility without agglomeration when the mass ratio of GR: Fe_3_O_4_ is 8:1. As the loading content of Fe_3_O_4_ increases, Fe_3_O_4_ particles begin to agglomerate on GR, but it is not obvious.

### 3.2. Thermal Properties and Electrical Insulation of Composites

**Figure 7:**
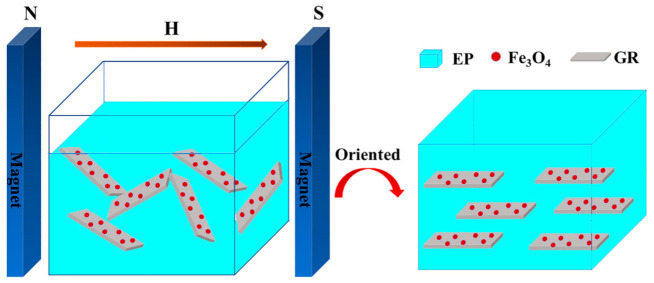
Schematic diagram of magnetic GR orientation under the induction of the magnetic field.

As shown in Figure 7, when Fe_3_O_4_ is loaded on the GR, under the induction of the magnetic field, Fe_3_O_4_ pulls the GR to spread along the direction parallel to the magnetic field, so that the magnetic GR can be directionally arranged in the EP matrix. The thermal conductivity of pure EP is 0.1810 W/mK, while the thermal conductivity of Fe_3_O_4_ doped EP is 0.1811 W/mK, indicating that Fe_3_O_4_ does not affect the thermal conductivity of EP. The specific thermal conductivity data have been listed in Table 2. In this experiment, when the content of GR was 0.134 wt.%, the thermal conductivity of composites reached 0.2801 W/mK, which was 54.8% higher than that of pure EP, that is, the thermal conductivity of the composites was about 1.55 times that of pure EP. In related studies, the content of GR in the composites was very large, generally more than 1 wt.%. For example, adding 10 wt.% GR in EP could improve the thermal conductivity to 1.53 W/mK [30], increasing the thermal conductivity by 8.5 times. However, the content of GR in EP was 74.6 times higher than that in this study. Moreover, the high GR content would also bring a series of problems, such as increase in cost, degradation of electrical insulation performance, and so on. Considering that the EP composite are mainly used in electronic packaging, it is necessary to add the GR as little as possible to ensure its electrical insulation.

**Figure 8:**
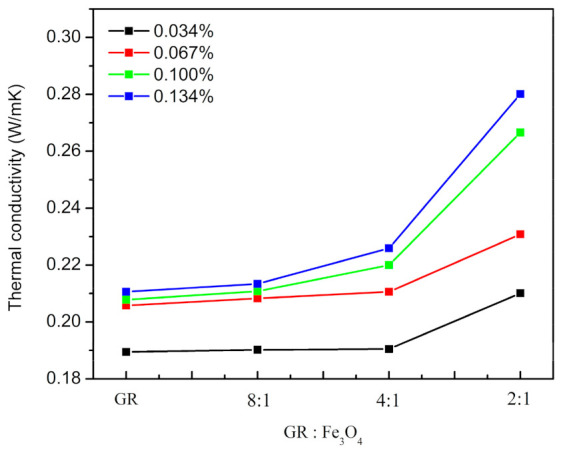
Thermal conductivity of EP/GR-Fe_3_O_4_ composites.

As shown in Figure 8, on the premise of the same degree of GR orientation, that is, the mass ratio of GR and Fe_3_O_4_ remains unchanged, the thermal conductivity increases gradually with the increase of GR content. This can be inferred from the fact that smaller content of GR tend to be wrapped by the EP and isolated from each other. With the increase of GR content, the heat transfer pathway is formed in the EP and the thermal conductivity increases.

When the mass ratio of GR to Fe_3_O_4_ is 8:1 and 4:1, the content of Fe_3_O_4_ is so small that GR cannot be arranged in a regular way, which results in a slight increase in thermal conductivity. When the mass ratio is 2:1, the thermal conductivity increases significantly. Therefore, it can be concluded that when the relative content of Fe_3_O_4_ in magnetic GR is small, the degree of directional arrangement of magnetic GR is low and it is difficult to obtain a high thermal conductivity. Once the mass ratio reaches 2:1, the GR is arranged regularly by the rotation of Fe_3_O_4_, and its high in-plane thermal conductivity can be fully utilized. Therefore, the thermal conductivity of the composites significantly improved. Next, the dynamic thermodynamic properties, thermal stability and electrical insulation of EP and composites (when the mass ratio of GR to Fe_3_O_4_ is 2:1) are studied.

**Figure 9:**
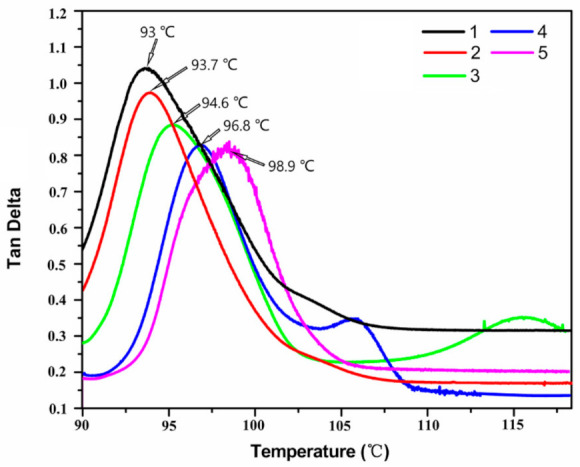
Dynamic thermomechanical analysis spectra of composites as a function of temperature with different magnetic GR contents. Curves 1–5 indicate that the mass fraction of magnetic GR in EP is 0.00%, 0.05%, 0.10%, 0.15%, and 0.20%, respectively.

The loss tangent (Tan δ) is an important parameter in the dynamic thermomechanical properties, which is sensitive to all molecular motions in the polymer, and the temperature corresponding to its peak can be used as the glass transition temperature (Tg) of the composites [31]. Figure 9 shows the relationship of Tan δ for EP and its composites as a function with temperature. It can be seen from the peak shift of the curves that the Tg of the composites are higher than that of pure EP, and the Tg gradually increases with the increase of GR content. This is attributed to the restraint of magnetic GR on the segmental motion of epoxy near the organic/inorganic interface [32], resulting in better dynamic thermomechanical properties for composites than pure EP.

**Figure 10:**
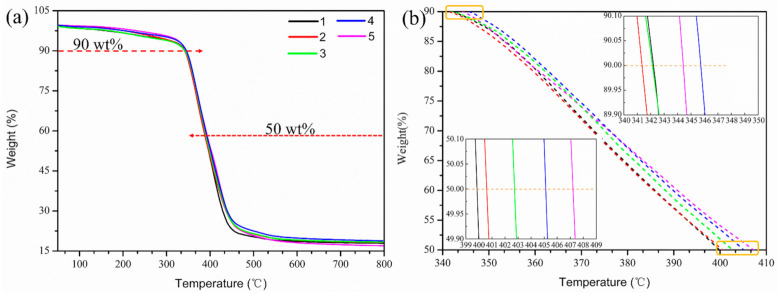
TGA curves of pure EP and EP/GR-Fe_3_O_4_ composites. Curves 1–5 indicate that the mass fraction of magnetic GR in EP is 0.00%, 0.05%, 0.10%, 0.15%, and 0.20%, respectively. (**a**) Overview of TGA curves; (**b**) magnified curves between 90 wt.% and 50 wt.%).

Thermal stability of the pure EP and EP/GR-Fe_3_O_4_ composites was studied by TGA. Figure 10a shows the TGA of pure EP and EP/GR-Fe_3_O_4_ composites with different mass fractions of magnetic GR. It can be seen that all composites show similar thermal decomposition curves as pure EP, indicating that the content of magnetic GR does not affect the original thermal decomposition mechanism of the EP matrix [8]. Magnifying the curves between 90% and 50% weight (Figure 10b), the curves of EP/GR-Fe_3_O_4_ composites decrease more slowly than that of EP. As shown in Table 3, the temperature for 10% weight loss (T_10%_) and 50% weight loss (T_50%_) were found that T_10%_ of EP and EP/GR-Fe_3_O_4_ are similar (the maximum difference is 3.2 °C), while T_50%_ have a great difference (the maximum difference is 8.4 °C) between them. Compared with pure EP, the addition of magnetic GR delayed the thermal decomposition process of EP. On the one hand, GR pieces effectively restrict the thermal movement of epoxy chain segments during combustion. On the other hand, GR pieces have a high thermal capacity and thermal conductivity. The synergistic effect of the two factors delays the thermal degradation of composites, making its thermal stability better than that of pure EP [33,34].

**Figure 11:**
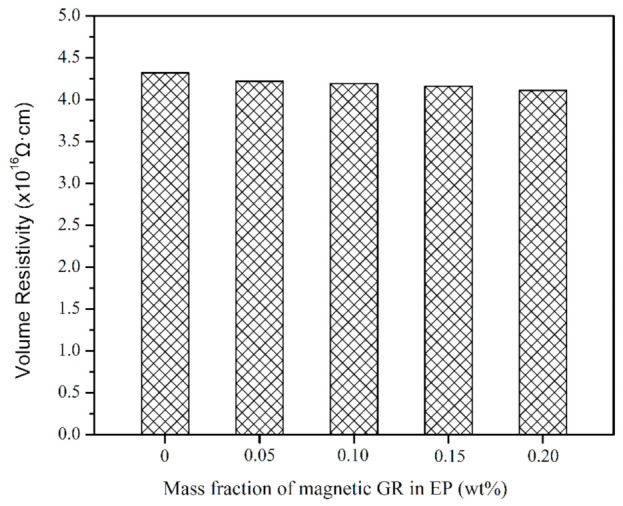
Volume resistivity of EP and EP/GR-Fe_3_O_4_ composites.

As for thermally conductive composites, the electrical insulation property is also a key factor in electronic packaging. Materials with high electrical insulation can ensure the safety and stability of electronic components [34]. Figure 11 shows the volume resistivity of EP and EP/GR-Fe_3_O_4_ composites as a function of magnetic GR content at 250 V. The volume resistivity of the composites has little difference compared with that of pure EP, which is kept at the same order of magnitude (𝗑 10^16^ Ω·cm) as that of pure EP. Moreover, none of the composites showed dielectric breakdown at the highest voltage (1000 V). Therefore, the thermal conductivity of the composites is improved on the premise of ensuring electrical insulation.

## 4. Conclusions

Using the chemical bonding method, GR and Fe_3_O_4_ were modified with functional groups, and the magnetic GR was obtained by stably loading Fe_3_O_4_ particles on GR sheets. The thermal properties of EP and EP/GR-Fe_3_O_4_ composites were further studied. At a constant mass ratio of GR: Fe_3_O_4_, the thermal conductivity increases gradually with the increase of GR content. When the amount of Fe_3_O_4_ loaded on GR is not sufficient, the orientation distribution of magnetic GR is relatively low, which results in a slight increase in thermal conductivity. When the mass ratio of GR to Fe_3_O_4_ reaches 2:1, the degree of GR orientation is significantly improved, and its high in-plane thermal conductivity can be fully utilized, resulting in the significant improvement of thermal conductivity. The dynamic thermomechanical properties and thermal stability of the EP/GR-Fe_3_O_4_ composites under this mass ratio are better than pure EP. Moreover, the addition of GR did not reduce the electrical insulation evidently of this composite.

## Figures and Tables

**Table 1:** The mass fraction of magnetic graphene (GR) in epoxy resin (EP).

Mass Fraction of GR in EP (wt.%)	Mass Fraction of Magnetic GR in EP (wt.%)
0.034	0.038 (a)
	0.040 (b)
	0.050 (c)
0.067	0.075 (a)
	0.080 (b)
	0.100 (c)
0.100	0.113 (a)
	0.125 (b)
	0.150 (c)
0.134	0.150 (a)
	0.167 (b)
	0.200 (c)

(a) Magnetic GR with mass ratio of GR to Fe_3_O_4_ of 8:1, (b) Magnetic GR with mass ratio of GR to Fe_3_O_4_ of 4:1, (c) Magnetic GR with mass ratio of GR to Fe_3_O_4_ of 2:1.

**Table 2:** Thermal conductivity (TC) of pure EP and EP/GR-Fe_3_O_4_ composites.

	TC (W/mK)	Only GR	GR: Fe_3_O_4_/8:1	GR: Fe_3_O_4_/4:1	GR: Fe_3_O_4/_2:1
The Mass Fraction of GR in EP(wt.%)					
0.034		0.1895 (±0.0003)	0.1902 (±0.0001)	0.1905 (±0.0004)	0.2101 (±0.0002)
0.067		0.2058 (±0.0004)	0.2083 (±0.0004)	0.2106 (±0.0002)	0.2308 (±0.0003)
0.100		0.2078 (±0.0004)	0.2108 (±0.0005)	0.2200 (±0.0003)	0.2666 (±0.0002)
0.134		0.2106 (±0.0003)	0.2134 (±0.0002)	0.2259 (±0.0003)	0.2801 (±0.0004)

**Table 3:** Thermal properties of pure EP and EP/GR-Fe_3_O_4_ composites.

The Mass Fraction of GR-Fe_3_O_4_ in EP (wt.%)	Tg (°C)	T_10%_ (°C)	T_50%_ (°C)
0	93.0	342.4	399.2
0.05	93.7	341.6	400.8
0.10	94.6	342.0	402.8
0.15	96.8	345.6	405.6
0.20	98.9	344.4	407.6

## Data Availability

The data presented in this study are available upon request from the corresponding author.
